# Points to consider: efficacy and safety evaluations in the clinical development of ultra-orphan drugs

**DOI:** 10.1186/s13023-017-0690-5

**Published:** 2017-08-23

**Authors:** Kojiro Maeda, Masayuki Kaneko, Mamoru Narukawa, Teruyo Arato

**Affiliations:** 1 Department of Regulatory Science, Hokkaido University Graduate School of Medicine, Sapporo, Japan; 2 Department of Clinical Medicine (Pharmaceutical Medicine), Graduate School of Pharmaceutical Science, Kitasato University, Tokyo, Japan; 3 Hokkaido University Hospital Clinic Research and Medical Innovation Center, Sapporo, Japan

**Keywords:** Ultra-orphan drugs, Review reports, Clinical trials, Guidance

## Abstract

**Background:**

The unmet medical needs of individuals with very rare diseases are high. The clinical trial designs and evaluation methods used for ‘regular’ drugs are not applicable in the clinical development of ultra-orphan drugs (<1000 patients) in many cases. In order to improve the clinical development of ultra-orphan drugs, we examined several points regarding the efficient evaluations of drug efficacy and safety that could be conducted even with very small sample sizes, based on the review reports of orphan drugs approved in Japan.

**Results:**

The clinical data packages of 43 ultra-orphan drugs approved in Japan from January 2001 to December 2014 were investigated. Japanese clinical trial data were not included in the clinical data package for eight ultra-orphan drugs, and non-Japanese clinical trial data were included for six of these eight drug. Japanese supportive data that included retrospective studies, published literature, clinical research and Japanese survey results were clinical data package attachments in 22 of the 43 ultra-orphan drugs. Multinational trials were conducted for three ultra-orphan drugs. More than two randomized controlled trials (RCTs) were conducted for only 11 of the 43 ultra-orphan drugs. The smaller the number of patients, the greater the proportion of forced titration and optional titration trials were conducted. Extension trials were carried out for enzyme preparations and monoclonal antibodies with high ratio. Post-marketing surveillance of all patients was required in 36 of the 43 ultra-orphan drugs.

For ultra-orphan drugs, clinical endpoints were used as the primary efficacy endpoint of the pivotal trial only for two drugs. The control groups in RCTs were classified as follows: placebo groups different dosage groups, and active controls groups. Sample sizes have been determined on the basis of feasibility for some ultra-orphan drugs.

We provide “Draft Guidance on the Clinical Development of Ultra-Orphan Drugs” based on this research.

**Conclusions:**

The development of ultra-orphan drugs requires various arrangements regarding evidence collection, data sources and the clinical trial design. We expect that this draft guidance is useful for ultra-orphan drugs developments in future.

## Background

Drugs have been developed mainly for diseases that target many individuals (such as lifestyle-related diseases), but the development of drugs that target rare diseases (i.e., orphan drugs) has been increasing [1, 2]. The clinical trial design and evaluation methods used for ‘regular’ (non-orphan) drugs are not applicable in the clinical development of orphan drugs in many cases because the numbers of targeted patients are quite limited, and the pathophysiology of the disease may not be known. The small size of the market for orphan drugs also hinders these drugs’ development.

Measures have been taken to promote orphan drug development in the European Union (EU) and the U.S. [3, 4] The number of drugs designated as the orphan drugs increases every year [5–8]. In Japan, the Ministry of Health, Labour and Welfare (MHLW) and the Pharmaceutical and Medical Devices Agency (PMDA) have collaborated to implement various promotion enterprises for orphan drug development [9]. The number of drugs designated as the orphan drugs in Japan has increased [8] but is still small compared to the numbers in the EU and U.S. [10] The European Medicines Agency (EMA) issued an orphan drug guideline in 2006 [11], and the U.S. Food and Drug Administration (FDA) created similar draft guidance in 2015 [12]. Japan has no equivalent guidance regarding orphan drugs.

In the U.S., the number of patients used for the designation of orphan drugs is <200,000; that is, 1 in 1500 people [12]. In the EU, the number of patients used for the designation of orphan drugs is ≤5 per 10,000 people, i.e., 1 in 2000 people; [13] in actual terms for some orphan drugs the number is often less than 1 per 100,000 people [14]. In Japan, the designation for orphan drugs targets diseases with <50,000 patients [15], which corresponds to 1 in 2600 people. Health Science Council of MHLW defines ultra-orphan drugs as pharmaceuticals for diseases that affect <1000 people [16]. The unmet medical needs of the individuals with very rare disease are increasing, to the point where a rare-disease patient group submitted “A request for ultra-orphan drug development support and drug discovery/intractable disease measures in Japan” [17].

Even in orphan drugs, it is required for approval to confirm the efficacy and safety in clinical trials like ‘regular’ drugs. But because of very few patients, the clinical trial designs and evaluation methods used for regular drugs are not applicable in the clinical development of orphan drugs in many cases.

Since ultra-orphan drugs are used by fewer patients than orphan drugs, it is assumed that development of ultra-orphan drugs needs various arrangements the clinical data package and clinical design, compared with orphan drugs other than ultra-orphan drugs.

Against this background, we examined the existing recommendations for the efficient assessments of the efficacy and safety of ultra-orphan drugs in clinical trials (even those with very small sample sizes). We also provide guidance for the promotion of ultra-orphan drug development.

## Methods

We obtained review reports of orphan drugs that were approved in Japan in the period from January 2001 to December 2014 from the PMDA website (http://www.pmda.go.jp/PmdaSearch/iyakuSearch/), and we investigated the clinical data packages of the drugs. When the information provided by the review reports was insufficient, we referred to the summary of application data available on the PMDA website. Information about the number of patients for target drugs in Japan was obtained from the proceedings of the Pharmaceutical Affairs and Food Sanitation Council Special Committee, in addition to the review reports. We investigated attachment of Japanese and non-Japanese data, randomized controlled trials (RCTs), dose-response trials, extension trials and post-marketing surveillances as the constitution of clinical data package, and efficacy end points, control arms and target sample sizes setting as the design of pivotal trials,

The data of clinical trials with healthy volunteers and those for other approved indications were not treated as evaluation data. The anticancer drug Phase I trials with solid-tumor patients were classified as evaluation data. Multinational trials including Japanese patients were treated as Japanese clinical trials. Trials investigating the number of doses and the method of administration were treated as dose-response trials.

## Results

### The backgrounds of the drugs examined

From January 2001 to December 2014, 156 orphan drugs were approved by the MHLW in Japan. We investigated 131 of those 156 drugs, excluding 17 anti-HIV drugs which are eligible for prior assessment based on the Pharmaceutical and Medical Safety Bureau (PMSB)/ELD Notification No. 1015 (dated November 12, 1998), and eight pandemic influenza vaccines. Forty-three ultra-orphan drugs and 88 orphan drugs (i.e., not ultra-orphan drugs) comprised the 131 drugs examined herein. The classification of these drugs is shown in Table 1.

**Table 1:** Classification of drugs examined

	Number of drugs (%)	
	Ultra-orphan drugs *n* = 43	Orphan drugs other than ultra-orphan drugs *n* = 88
Therapeutic category		
Metabolic drug	16 (37%)	12 (14%)
Anticancer drug	11 (26%)	33 (38%)
Biological drug	8 (19%)	7 (8%)
Cardiovascular drugs	-	10 (11%)
Central nervous system drugs	1 (2%)	6 (7%)
Sensory organ agents	-	5 (6%)
Hormones	1 (2%)	4 (4%)
Peripheral nervous system drugs	1 (2%)	3 (3%)
Digestive organ agents	1 (2%)	2 (2%)
Others	4 (10%)	6 (7%)
Drugs with new ingredients		
	31 (72%)	50 (57%)
Approved in other countries outside Japan		
	36 (84%)	63 (72%)

Classification of therapeutic category, drugs with new ingredients and drugs that had already been approved in countries outside Japan of the 43 ultra-orphan drugs and 88 orphan drugs other than ultra-orphan drugs approved in Japan in the period from January 2001 to December 2014. The classification of drug’s therapeutic category was based on the standard commodity numbers used in Japan

### The composition of the clinical data packages

In general, Japanese clinical trials data are required for the approval of regular drugs in Japan. But in ultra-orphan drugs, very limited Japanese patients’ data alone may be insufficient to explain the efficacy and safety; therefore, we investigated the usage of non-Japanese clinical data as an attachment in the clinical data package.

**Fig. 1:**
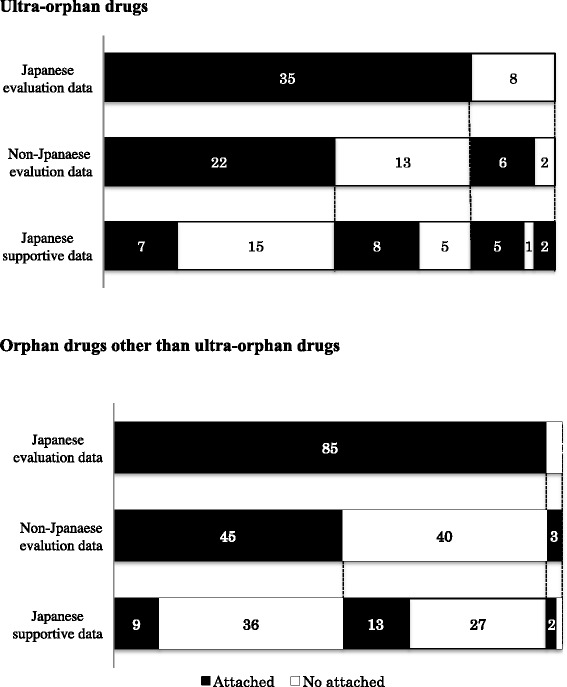
Composition of data package. Forty-three ultra-orphan drugs and 88 orphan drugs other than ultra-orphan drugs approved in Japan from January 2001 to December 2014. The numbers in the figure are the numbers of drugs

For 35 of the 43 ultra-orphan drugs (81%), Japanese clinical trial data were included in the clinical data packages as evaluation data (Fig. 1). For each therapeutic category, Japanese clinical trial data were provided as an attachment in all eight of the biological drugs, 10 of the 11 anticancer drugs and 12 of the 16 metabolic drugs. Japanese clinical trial data were not included as an attachment in the clinical data package for eight ultra-orphan drugs, and non-Japanese clinical trial data were included as a clinical data package attachment for six of these eight drugs (Fig. 1). These six drugs were classified as four metabolic drugs, an anticancer drug and a respiratory-organs agent. Clinical trial data were not attached for two drugs (imatinib [brand name: Gleevec; indications: eosinophilic leukocytosis, chronic eosinophilic leukemia] and thalidomide [brand name: Thaled; indication: erythema nodosum leprosum]), which were approved based on the effectiveness and safety of the product already in the public domain in the medical and pharmaceutical fields.

Japanese supportive data that included retrospective studies, published literature, clinical research (non-PMDA authorized trials) and Japanese survey results were clinical data package attachments in 22 of the 43 ultra-orphan drugs (51%) (Fig. 1). Japanese supportive data were attached in seven of the 11 anticancer drugs (64%), and the data for other cancer indication attached as supportive data were mainly used for safety evaluation. In our present analysis, we defined the number of patients who are the target for an ultra-orphan drug as <1000. To determine the features of the clinical data packages of smaller patient populations, we examined the cases in which the number of patients was <500 and those in which the number of patients was <100. As the number of patients decreased, the ratio of drugs with Japanese clinical trial data decreased, and the ratio of drugs with non-Japanese clinical trial data or Japanese supportive data increased.

In general, when non-Japanese trial data are used for regulatory submission, the efficacy and safety data of Japanese and non-Japanese patients are usually compared in order to provide a rationale for the extrapolation of the data. However, for risedronate (brand name: Actonel/Benet, indication: Paget’s disease) and sapropterin (brand name: Biopten, indication: hyper-phenylalaninemia), pharmacokinetic data of healthy Japanese and non-Japanese adults were used for extrapolation of the data.

Regarding the orphan drugs other than ultra-orphan drugs examined herein (these drugs will hereafter be referred to as simply “orphan drugs” in this publication; this excludes ultra-orphan drugs), the clinical trial data were included for all 88 drugs and Japanese clinical trial data were attached as evaluation data in 85 of these 88 drugs (97%) (Fig. 1). For each therapeutic category, Japanese clinical trial data were included for all of the metabolic drugs and for 31 of the 33 anticancer drugs (94%). The ratios of both the ultra-orphan drugs and the orphan drugs for which Japanese clinical trial data were included were higher in the metabolic drugs and anticancer drugs than the other types of drugs. Japanese clinical trial data were not included for three orphan drugs approved for additional indications.

### Multinational trials

Multinational trials were conducted for three ultra-orphan drugs: ruxolitinib (brand name: Jakavi, indication: myelofibrosis), elosulfase alfa (brand name: Vimizim, indication: mucopolysaccharidosis IVa) and sirolimus (brand name: Rapalimus, indication: lymphangioleiomyomatosis). All three of these drugs were approved in Japan in 2014. Multinational trials were also conducted for 11 of the 88 orphan drugs (13%); seven of these 11 drugs are anticancer drugs.

### Randomized controlled trials

**Fig. 2:**
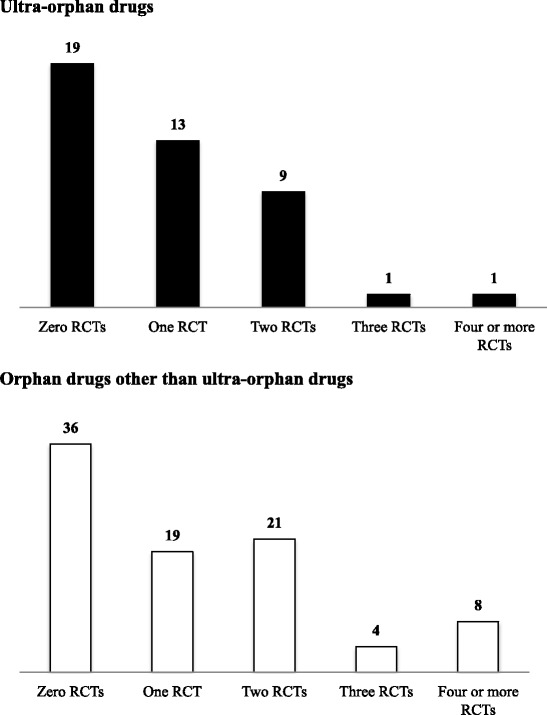
The numbers of RCTs conducted. *Upper panel*: Ultra-orphan drugs. *Lower panel*: Orphan drugs other than ultra-orphan drugs. The numbers in the figure are the numbers of drugs. RCTs: randomized controlled trials

In order to ensure the reliability of the results, it would be desirable, in principle, for the efficacy to have been confirmed in two or more RCTs. More than two RCTs were conducted for only 11 of the 43 ultra-orphan drugs (26%) and for 33 of the 88 orphan drugs (38%). The ratio of drugs that RCTs were conducted was thus lower in the ultra-orphan drugs compared to the orphan drugs (Fig. 2).

More than one RCT was conducted in 24 of the 43 ultra-orphan drugs (56%) and 52 of the 88 orphan drugs (59%); these percentages are not significantly different. However, the number of RCTs conducted in Japan specifically was much lower in the ultra-orphan drugs (three of 43, 7%) compared to the orphan drugs (30 of 88, 34%) (Table 2). Regarding the therapeutic categories, RCTs were conducted in 12 of the 16 metabolic drugs (75%), five of the 11 anticancer drugs (45%), and three of the eight biological drugs (37%), with the highest RCT rate in the metabolic drugs.

**Table 2:** Clinical trials conducted for ultra-orphan drugs and orphan drugs

	RCT (number of drugs)		Dose-response trial (number of drugs)						Extension trials (number of drugs)
	Japan	Outside of Japan	Japan			Outside of Japan			
			Parallel-group trial and traditional 3 + 3 design	Forced titration trial	Optional titration trial	Parallel-group trial and traditional 3 + 3 design	Forced titration trial	Optional titration trial	
All ultra-orphan drug	3/43 (7%)	21/43 (49%)	5/43 (12%)	4/43 (3%)	13/43 (30%)	16/43 (37%)	2/43 (5%)	8/43 (19%)	16/43 (37%)
Metabolic drug	1/16 (6%)	11/16 (69%)	1/16 (6%)	1/16 (6%)	6/16 (38%)	7/16 (44%)	1/16 (6%)	4/16 (25%)	10/16 (63%)
Anticancer drug	1/11 (9%)	4/11 (36%)	4/11 (36%)	0/11 (0%)	4/11 (36%)	6/11 (55%)	0/11 (0%)	2/11 (18%)	1/11 (9%)
Biological drug	1/8 (13%)	2/8 (25%)	0/8 (0%)	2/8 (25%)	2/8 (25%)	1/8 (13%)	1/8 (13%)	1/8 (13%)	2/8 (25%)
Others	0/8 (0%)	4/8 (50%)	0/8 (0%)	1/8 (13%)	1/8 (13%)	2/8 (25%)	0/8 (0%)	1/8 (13%)	3/8 (38%)
All orphan drugs other than ultra-orphan drugs	30/88 (34%)	41/88 (47%)	33/88 (38%)	1/88 (1%)	31/88 (35%)	30/88 (34%)	0/88 (0%)	13/88 (15%)	8/88 (9%)
Metabolic drug	8/12 (7%)	3/12 (25%)	5/12 (42%)	0/12 (0%)	7/12 (58%)	3/12 (25%)	0/12 (0%)	3/12 (25%)	2/12 (17%)
Anticancer drug	7/33 (21%)	9/33 (27%)	18/33 (55%)	1/33 (3%)	5/33 (15%)	11/33 (33%)	0/33 (0%)	5/33 (15%)	2/33 (6%)
Biological drug	3/7 (43%)	1/7 (14%)	2/7 (29%)	0/7 (0%)	3/7 (43%)	0/7 (0%)	0/7 (0%)	1/7 (14%)	0/7 (0%)
Others	12/36 (33%)	28/36 (85%)	18/36 (50%)	0/36 (0%)	16/36 (44%)	17/36 (47%)	0/36 (0%)	4/36 (11%)	4/36 (11%)

Classification of clinical trials of the ultra-orphan drugs and orphan drugs other than ultra-orphan drugs for each therapeutic category. Others: drugs other than metabolic drugs, anticancer drugs and biological drugs

Three ultra-orphan drugs in which RCTs were conducted in Japan were elosulfase alfa, freeze-dried sulfonated human normal immunoglobulin (brand name: Kenketsu Venilon-I, indications: Churg-Strauss syndrome), and sirolimus. One RCT of sirolimus and two RCTs of elosulfase alfa were multinational trials. The numbers of Japanese patients with the disease targeted by the sirolimus and elosulfase alfa were very low (<500 and <100, respectively); however, multinational trials still provide the opportunity to conduct RCTs even for ultra-orphan drugs.

Dosing-period randomized, double-blind, placebo-controlled, cross-over trial was conducted in Japan for freeze-dried sulfonated human normal immunoglobulin, which was developed only in Japan. In this trial, some treatment periods including active drug or placebo treatment periods were set up, and the drug were randomized in each treatment period. In its review report, the PMDA stated that, “When a subjective endpoint must be used in the rare-disease area, it is useful for drug development to use such a trial design” [18].

### Dose-response trials

Dose-response trials including parallel-group dose-response trials, traditional 3 + 3 design, forced titration and optional titration trials were conducted for 35 of the 43 ultra-orphan drugs (81%) and 78 of the 88 orphan drugs (89%), with a lower rate of dose-response trials conducted in Japan for ultra-orphan drugs compared to orphan drugs.

**Fig. 3:**
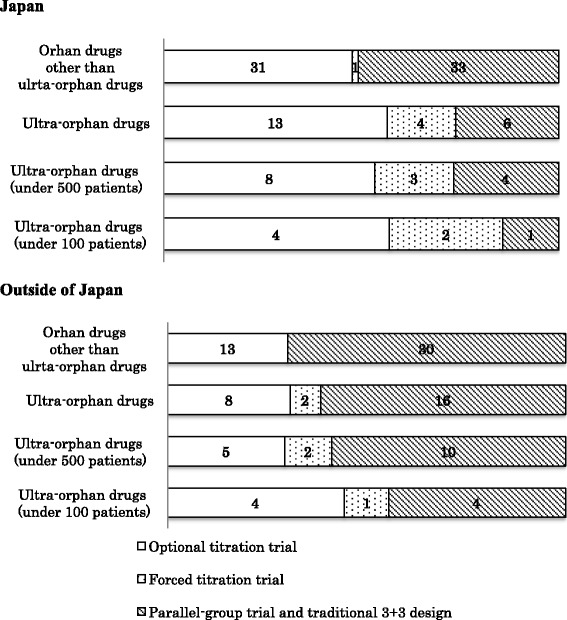
Classification of dose-response trials. *Upper panel*: Classification of the dose-response trials conducted in Japan. *Lower panel*: Classification of the dose-response trials conducted outside of Japan. The numbers in the figure are the numbers of drugs

When some of the above-mentioned dose-response trials were conducted for a drug, the trials were classified according to clear order as the basis for dose-setting in the order of i) parallel-group trials and traditional 3 + 3 design, ii) forced titration trials, and iii) optional titration. The smaller the number of patients, the greater the proportion of forced titration and optional titration trials were conducted both in and outside of Japan (Fig. 3). The ratio of drugs for which parallel-group trials and traditional 3 + 3 design were conducted was higher outside of Japan compare to in Japan for both ultra-orphan drugs and orphan drugs (Table 2).

Among the metabolic drugs, dose-response trials were conducted in 15 of the 16 ultra-orphan drugs. The ultra-orphan metabolic drug for which a dose-response trial was not conducted was sapropterin (brand name: Biopten, indication: hyper-phenylalaninemia), and its approval was for additional indications. For all metabolic orphan drugs, dose-response trials were conducted in Japan (Table 2).

Regarding the anticancer drugs, dose-response trials were conducted in 10 of 11 ultra-orphan drugs, with the exception of imatinib (indications: eosinophilic leukocytosis, chronic eosinophilic leukemia). The ratio of dose-response trials (especially parallel-groups dose response trials and traditional 3 + 3 design) conducted is higher for anticancer drugs compared to the other therapeutic categories both in and outside of Japan (Table 2).

The dosage and administration were determined based on only optional titration trials in some drugs. For tamibarotene (brand name: Amnolake, indication: acute myelogenous leukemia), the dosage was appropriately increased or decreased in clinical trials. Sufficient efficacy was obtained at the initial dose; therefore, the approved dose was the initial dose. For tetrabenazine (brand name: Choreazine, indication: Huntington’s disease), the approved maximum maintenance dose was set based on the efficacy at the highest dose in optional titration trials.

The dosage and administration of ultra-orphan drugs for which dose-response trials were not conducted were determined based on the results of pharmacology data and plasma concentrations in Phase I trials of healthy volunteers (tafamidis [brand name: Vyndaqel, indication: familial amyloid polyneuropathy]), the dosage and administration approved outside of Japan (hemin [brand name: Normosang, indication: acute porphyria]), and published literature and guidelines (drying polyethylene glycol treatment human immunoglobulin [brand name: Kenketu Glovenin-I, indications: Stevens-Johnson syndrome, toxic epidermal necrolysis]).

### Extension trials

Extension trials were carried out in 16 of the 43 ultra-orphan drugs (37%) and eight of the 88 orphan drugs (9%) (Table 2). Ten metabolic drugs and two biological drugs were among those 16 ultra-orphan drugs. In other words, the ratio of extension trials conducted was high for enzyme preparations and monoclonal antibodies.

In 12 of the above-mentioned 16 ultra-orphan drugs, patients who had already participated in a clinical trial were re-entered in an extension trial. For alglucosidase alfa (brand name: Myozyme, indication: glycogenosis Type 2), two types of extension trials were conducted: expanded-access studies in severely affected patients who failed to meet the inclusion criteria for clinical studies, and extended studies for the patients who had been treated in a past clinical trial with another company’s formulation. For canakinumab (brand name: Ilaris, indication: cryopyrin-associated periodic syndrome), some patients enrolled in phase II trials were re-entered in not only extension trials but also in phase III trials.

### Post-marketing surveillance

Clinical trial data generally have their limitations, and thus post-marketing surveillance is useful to complement a drug’s efficacy and safety data. Post-marketing surveillance has been required for all orphan drugs and ultra-orphan drugs in Japan. In addition, post-marketing surveillance of all patients was required in 36 of the 43 ultra-orphan drugs (84%) and 65 of the 88 orphan drugs (74%).

In addition, post-marketing clinical trials were required in three of the 43 ultra-orphan drugs (7%) and seven of the 88 orphan drugs (8%). For example, a post-marketing clinical trial was required in order to investigate the appropriate dosage of the ultra-orphan drug basiliximab (brand name: Simulect, indication: acute rejection after renal transplantation) for Japanese pediatric patients. For the drug agalsidase beta (brand name: Fabrazyme, indication: Fabry disease), a post-marketing clinical trial was required to confirm the efficacy and safety in patients with cardiac involvement in Fabry disease, which is an atypical variant of Fabry disease in men with left ventricular hypertrophy, for which clinical trials had not been conducted in Japan. As a result of the required post-marketing clinical trial, the wording “Efficacy and safety in cardiac involvement in Fabry disease patients have not been established” as a Precaution was deleted from the drug’s package insert.

### Efficacy endpoints

In general, true endpoints should be used as the primary efficacy endpoints of clinical trials. For ultra-orphan drugs, however, clinical endpoints were used as the primary efficacy endpoint of the pivotal trial for vemurafenib (brand name: Zelboraf, indication: malignant melanoma), in which the overall survival was used in a phase III trial and for alglucosidase alfa (brand name: Myozyme, indication: glycogen storage disease type II), ventilator-free survival at 18 months age was used in a phase II/III trial (Table 3).

**Table 3:** Comparison of clinical trials design of ultra-orphan drugs

	Anticancer drug *n* = 11	Metabolic drug *n* = 16	Others *n* = 16
Efficacy endpoint (Example)			
True endpoint	• Overall survival (vemurafenib)	• Ventilator-free survival (alglucosidase alfa)	
Surrogate endpoint	• Major CyR (dasatinib) • Reduction in spleen volume (ruxolitinib) • Forced expiratory volume 1 s (sirolimus)	• The 12-min walking distance (galsulfase) • Blood alkaline phosphatase (risedronate) • Blood phenylalanine (sapropterin)	• Manual muscle test (freeze-dried sulfonated human normal immunoglobulin) • Haemoglobin stabilization (eculizumab) • Transcranial sonography (tetrabenazine)
Controls (RCT)			
Placebo	3/7 trials	13/17 trials	11/14 trials
Active controls	3/7 trials	1/17 trials	1/14 trials
Different dosage	1/7 trials	3/17 trials	2/14 trials
Number of patients (Min-Max)			
RCT	89–675	6–176	18–965
Single-arm trial	6–724	1–168	1-7252^a^
Statistical evidence (RCT)			
Significance level	5%	5%	5%
Power	80–90%	80–90%	80–90%

Classification of clinical trials designs of the ultra-orphan drugs for each therapeutic category. Others: drugs other than metabolic drugs, anticancer drugs

^a^ Study on preventive administration of mefloquine and next was 168 patients of eculizumab

When surrogate endpoints are used, it is desirable that surrogate endpoints reflect true endpoints. However, for galsulfase (brand name: Naglazyme, indication: mucopolysaccharidosis type IV), it was difficult to evaluate the clinical efficacy endpoints (survival and disease progression) in clinical trials because of limited patient populations and varying progression rates of the disease. Therefore, the 12-min walking distance was used as the primary efficacy endpoint even though the relationship between this endpoint and the clinical endpoints was unclear. For alglucosidase alfa, the efficacy endpoints were different in every trial. PMDA requested an explanation of the relationship between these efficacy endpoints and the clinical endpoints, and the correlation with the true endpoints was revealed by an analysis of a natural history study.

For some metabolic drugs, the primary efficacy endpoints themselves could not be set. For example, it was impossible to determine the appropriate efficacy endpoint in the pivotal phase II trial of miglustat (brand name: Brazaves, indication: Niemann-Pick disease type C [NPC]) because this was the world’s first comparative trial of a therapeutic drug for NPC, and no guidelines were available. The primary efficacy endpoint was therefore not set, and efficacy was evaluated by collecting as many efficacy endpoints as possible. For canakinumab, the definition of the primary efficacy endpoint of the phase II trial was changed following a discussion between the FDA and EMA based on the interim analyzed data, since there were no clinical trial experience and no established efficacy endpoints. The changed efficacy endpoint was then used in a phase III trial and a long-term study. Thus, in some drugs, efficacy endpoints have been examined in an exploratory manner in phase II trials.

Some ultra-orphan drugs, especially metabolic drugs, were approved even though no statistically significant difference was observed in the primary efficacy endpoints of RCTs. For agalsidase alfa (brand name: Replagal, indication: Fabry disease), a statistically significant difference was not observed in the Brief Pain Inventory score or the serum level of ceramide trihexoside as the primary efficacy endpoint of phase II trials. However, significant differences were observed in several secondary efficacy endpoints in these trials. In addition, significant differences were observed in two of the three primary efficacy endpoints in this drug’s phase II open label trial in Japan; therefore, it was judged to be effective as a whole and was approved by the PMDA. For laronidase (brand name: Aldurazyme, indication: mucopolysaccharidosis Type I), two primary efficacy endpoints were used in a phase III trial. A significant difference was observed in forced vital capacity after 26 weeks, but it was not observed in the 6-min walk distance even though a positive trend was observed. The efficacy of laronidase was then evaluated based on two primary efficacy endpoints and 16 secondary efficacy endpoints which could reflect various types of clinical conditions. Laronidase was approved by the PMDA, because an evaluation system for the treatment of mucopolysaccharidosis type I was not established at that time. Thus, the efficacy of some drugs has been comprehensively evaluated by including secondary efficacy endpoints, with subsequent approval. In contrast, significant differences in the primary efficacy endpoints were observed in all of the 88 approved orphan drugs examined herein.

### Controls

Thirty-eight RCTs were conducted for 24 of the 43 ultra-orphan drugs. The control groups in those RCTs were classified as follows: placebo groups (27 trials), different dosage groups (six trials), and active controls groups (five trials).

For some ultra-orphan drugs, efficacy was evaluated in RCTs using reference drug groups for non-statistical drug comparisons. Phase II trials of cinacalcet (brand name: Regpara, indication: hypercalcemia), a placebo group (8 patients) was set as the reference group for a comparison with the 40 patients in the active group.

For some drugs, the results of clinical trials were compared with external controls. For ultra-orphan metabolic drug alglucosidase alfa, the result of phase II/III dose-response trial was compared with external control (matched 61 patients’ data were extracted from the epidemiological research data for 168 infant glucose storage disease II patients). For the orphan drug anti-human thymocyte immunoglobulin, rabbit (brand name: Thymogloblin, indication: graft versus host disease), the results of a Japanese phase II trial were compared with the data of patients from the Japan Society for Hematopoietic Cell Transplantation as an external control.

Regarding the evaluation of preventive effect, an intra-individual comparative trial was conducted to evaluate its effect against acute porphyria for the ultra-orphan drug hemin.

When the natural history of the target disease is clear and treatment effect is huge, single-arm trials with thresholds can be used for efficacy evaluations. A drug’s efficacy can be evaluated in single-arm trials when the desired therapeutic effects of the drug can be predicted on the basis of its known mechanism of action. Examples of this are the method of supplement of defective molecular (e.g., sodium phenylbutyrate [brand name: Buphenyl, indications: urea cycle disorders]) and the method of supplement of the substrate of the defective enzyme (e.g., betaine [brand name: Cystadane, indication: homocystinuria]).

### Determination of the sample sizes -statistical evidence-

Sample sizes of clinical trials were various even in ultra-orphan drugs (Table 3). Typically sample sizes are determined by using a significance level of 5% and a power of 80–90%. A significance level of 5% and a power of 80–90% were used for ultra-orphan drugs in which target sample sizes were determined with statistical evidence in RCTs, even if the number of patients was very limited (Tables 3 and 4). In contrast, the sample size was determined by using a significance level of 10% for rufinamide (brand name: Inovelon, indication: Lennox-Gastaut syndrome), which is an orphan but not ultra-orphan drug, since it was difficult to enroll enough patients.

**Table 4:** Evidence of sample sizes setting of pivotal trials

	Randomized controlled trials	Open trials
Target sample sizes with statistical evidence	Agalsidase alfa^a^ (26 patients) Canakinumab^a^ (31 patients) Galsulfase^a^ (39 patients) Agalsidase beta^a^ (58 patients) Risedronate^a^ (120 patients) Elosulfase alfa^a^ (176 patients) Sunitinib ^b^ (191 patients) Tafamidis^c^ (125 patients) Basiliximab^c^ (376 patients) Dornase alpha^c^ (968 patients)	Cinacalcet^a^ (46 patients) *Tamibarotene* ^b^ (39 patients) Nilotinib^b^ (282 patients)
Feasibility	Idursulfase^a^ (96 patients) Dasatinib^b^ (150 patients) Eculizumab^c^ (87 patients)	Phenylbutyrate^a^ (11 patients) Hemin^b^ (2 patients) Vorinostat^b^ (6 patients) Cladribine^b^ (9 patients) *Freeze-dried concentrated human activated protein C* ^c^ (3 patients) *Metreleptin* ^c^ (4 patients) *Drying polyethylene glycol treatment human immunoglobulin* ^c^ (7 patients) *Infliximab* ^c^ (Behcet’s disease) (12 patients) *Tocilizumab* ^c^ (Ewing sarcoma) (28 patients)

Classification according to trial design/case setting basis in the pivotal trials of the ultra-orphan drugs. ^a^Metabolic drugs, ^b^Anticancer drugs, ^c^drugs other than metabolic drugs and anticancer drugs. (patients) behind the drug name: real sample sizes (when there were controls, the sample sizes of the total of controls and this drug is given). *Italics* were not approved outside of Japan at the time of Japanese approval

Sample sizes have also been determined on the basis of feasibility without a consideration of statistical power even when RCTs were conducted for some drugs (Table 4). For example, the enforceable maximum sample size was used as the target sample size in the phase II/III trial of idursulfase (brand name: Elaprase, indication: mucopolysaccharidosis II). It was difficult to calculate the target sample size because the efficacy endpoint was a composition score, and because of the very limited patient population.

In single-arm trials, a significance level of 5% and a power of 80–90% were used for drugs in which target sample sizes were determined based on statistical evidence, like the RCTs. On the other hand, the sample sizes were determined on ethe basis of feasibility in some drugs (e.g. metreleptin [brand name: Metreleptin/Myalept, indication: lipoatrophy]) (Table 4).

### The draft guidance

We investigated the clinical data packages of ultra-orphan drugs approved in Japan in the period from January 2001 to December 2014, and we provide our recommendations for efficient evaluations of the efficacy and safety of ultra-orphan drugs (even those with very small sample sizes). We have devised the following “Draft Guidance on the Clinical Development of Ultra-Orphan Drugs” based on these recommendations.

“*Draft Guidance on the Clinical Development of Ultra-Orphan Drugs”*

#### Introduction

In the clinical development of ultra-orphan drugs for the treatment of very rare diseases, it is often difficult to conduct clinical trials that aim to confirm the efficacy and safety of the drugs. This is partly because very limited patient populations hinder the recruitment of a sufficient number of trial subjects. Another reason for the difficulty with clinical trials is that very limited patient populations also hinder the elucidation of pathologies of very rare diseases. For these reasons, clinical data packages of ultra-orphan drugs for regulatory submission should be prepared carefully and flexibly. Each clinical trial of an ultra-orphan drug needs various arrangements at each stage of the trial, such as the trial design, trial conduct, data analysis, and data interpretation. This guidance provides recommendations to efficiently evaluate the efficacy and safety of ultra-orphan drugs in clinical trials even with very small sample sizes.

In this guidance, an ultra-orphan drug is defined as a medicinal product for a very rare disease that affects less than 1000 individuals in Japan.

#### Evidence building

##### Randomized controlled trials

In general, one of the effective development strategies for showing high-level evidence of the efficacy and safety of a drug for regulatory approval is to show that the efficacy and safety of the drug are significantly higher than those of appropriate control drugs in two or more randomized controlled trials (RCTs). Japan’s Pharmaceutical and Medical Devices Agency (PMDA) states, “In order to ensure the reliability of the results, it would be desirable, in principle, for the efficacy to have been confirmed in two or more randomized controlled studies”, in their notification entitled “Points to Be Considered by the Review Staff Involved in the Evaluation Process of New Drug” (April 17, 2008) [19]. The notification also states, “Especially, for drugs in the field of orphan diseases or serious diseases for which existing therapies have not yet been established, final decisions should not be based exclusively on the points covered in this document, but should also take into consideration other points such as the clinical significance of the drug. Even for such drugs, however, the scientific evaluation using appropriate data should be based on a full understanding of the purpose and principle of this document”. This idea of clinical evaluation should be fully understood and based on the idea that the clinical development of ultra-orphan drugs should be conducted appropriately because the number of potential subjects is very limited.

When a sufficient number of patients with the target disease can be recruited for an ultra-orphan drug, two or more RCTs may be feasible, including dose-finding trials and non-Japanese trials. When a sufficient number of patients cannot be recruited, a single RCT or single-arm trials may be the only option for the evaluation of drug efficacy. When the desired therapeutic effects of a drug can be predicted on the basis of its known mechanism of action or when the natural history of the target disease is clear, it may be possible to evaluate the drug’s efficacy in single-arm trials. When this is not the case, it may be possible to evaluate the drug’s efficacy in RCTs using reference drugs (i.e., placebos or conventional drugs used for nonstatistical drug comparisons).

##### Dose-response trials

Dose-response trials are essential to identify the appropriate dose of a drug. The trials can use various designs such as a parallel-group trial, crossover trial, forced titration and optional titration trial. For ultra-orphan drugs as well as regular drugs, dose-response trials should be conducted whenever possible. For some very rare diseases, it may not be possible to conduct a dose-response trial with two or more dose groups because of an extremely small sample size. In such cases, an individual forced or optional dose-escalation trial in which each subject must or may receive different doses may be feasible for dose selection with a small sample size. However, such dose-escalation trials have limitations; for example, the trials are inapplicable for some very rare diseases. In addition, the carry-over effects of trial treatments may affect the drug evaluation.

When no dose-response trial is conducted, rationales for dosage selection should be provided on the basis of pharmacological effects, pharmacokinetic/pharmacodynamic trials in healthy adults, non-Japanese dose-response trials (if available), clinical research, drug use result surveys, published literature and other relevant data. When an application for an additional indication is planned, recommended doses for currently approved indications should be fully considered. When the rationales for dose selection are not clear, doses should be re-selected after marketing if necessary.

##### Extension trials

Extension trials are often useful for the clinical development of ultra-orphan drugs, including enzyme replacement therapy drugs and antibody drugs for long-term use. Extension trials can increase clinical data and be used to evaluate the long-term safety and efficacy of a drug. Extension trials may also enable subjects to continue the trial treatment.

#### Data sources

##### Multinational trials and non-Japanese trials

When clinical trials that can produce high-level evidence are not feasible in Japan because of a very limited number of Japanese patients with the target disease, one of the effective development strategies is the use of appropriately designed multinational trials (e.g., RCTs). Multinational trials can recruit more subjects than Japanese trials. For ultra-orphan drugs as well as regular drugs, multinational trials should be conducted with reference to the notification entitled “Basic Principles on Global Clinical Trials” (PFSB/ELD Notification No. 0928010, Director of the Evaluation and Licensing Division, Pharmaceutical and Food Safety Bureau, Ministry of Health, Labour and Welfare [MHLW], dated September 28, 2007).

Another effective development strategy is the use of non-Japanese trial data. When the target disease is very rare in Japan but not in other countries, the results of non-Japanese trials (including RCTs and dose-response trials) can be used for regulatory submission in Japan. When only single-arm trials are feasible in Japan, when no dose-response trials have been conducted in Japan, or when the number of Japanese clinical trials is not sufficient for regulatory submission, the results of non-Japanese trials are useful to increase the levels of evidence.

When the results of multinational or non-Japanese trials are used for regulatory submission in Japan, rationales should be provided for the extrapolability of efficacy and safety data from multinational or non-Japanese trials to the Japanese target population. When the sample sizes are too small to assess the extrapolability, pharmacokinetic, pharmacodynamic or biomarker data of Japanese or non-Japanese healthy adults can be used for extrapolation to the Japanese target population.

##### Clinical research and drug use result surveys

Another effective development strategy is the use of clinical research and drug use result surveys conducted in Japan. The results of such studies can be used as supportive data for regulatory submission in Japan. Although clinical research and drug use result surveys do not always provide high-level evidence or highly credible data, such studies provide clinical data under actual conditions of drug use in Japan. Similar data obtained in other countries can be used as well. Such data include data on clinical research, advanced medical technologies, compassionate use, case reports of off-label drug use, the published literature and treatment guidelines in Japan and other countries.

#### Designing pivotal trials

##### Efficacy endpoints

As with clinical trials of regular drugs, true endpoints should be used in the clinical trials of ultra-orphan drugs as the primary efficacy endpoints. However, surrogate endpoints may be helpful to reduce the sample size and the duration of a clinical trial. When any surrogate endpoints are used for an efficacy evaluation, an explanation should be provided regarding the relationship between the surrogate and true endpoints whenever possible.

For comprehensive efficacy evaluations, as many secondary efficacy endpoints should be used as possible, and their consistency and the relationships between the endpoints should be assessed.

##### Controls

The efficacy of ultra-orphan drugs can be evaluated in single-arm trials using no control drugs when the natural history of the target disease is clear. For some ultra-orphan drugs, however, RCTs using reference drugs may be more helpful to evaluate drug efficacy with a very small sample size. The necessity of concurrent controls should be assessed with consideration of the cause and nature (including the natural history) of the target disease, the mechanism of action of the drug, the efficacy endpoints to be used in the clinical trial, and the expected efficacy levels of the drug.

The results of epidemiologic research on the natural history of a target disease can be used as external controls. In this case, the subject characteristics of the epidemiologic research should be similar to those of the clinical trial (s). When an external control is used as a development strategy, matching should be actively used to minimize differences in subject characteristics between the external research and the clinical trial (s) and to increase the levels of evidence in the clinical trial. Concurrent controls should be used, whenever possible, to avoid any influence of different timings of observation between treatments for a drug’s evaluation. When a historical control is used, rationales for the use of the control should be provided. Patient registries are useful for finding appropriate external controls as well as for recruiting trial subjects efficiently.

##### Designs

When an ultra-orphan drug is compared with a control drug, a concurrently controlled parallel-group trial is usually considered. Another option is a crossover trial that can be conducted with a smaller sample size than that of a parallel-group trial. However, crossover trials are applicable to limited situations, and the carry-over effects of test treatments may bias the results. When the efficacy without a carry-over effect of the drug is assessed, dosing-period randomized, double-blind, placebo-controlled trials may be applicable to, for example, rare diseases that require subjective efficacy endpoints. Although such trials are not applicable to all diseases, they are one of the well-designed study types for obtaining high-level evidence even when the sample size is small. When the natural history of the target disease is clear, single-arm trials with thresholds can be used for the efficacy evaluation.

When preventive effects of an ultra-orphan drug are evaluated, intra-individual comparative trials can be used for the efficacy evaluation. In this case, the observation period and the treatment period should be set appropriately on the basis of the incidence of the target disease.

##### Determination of sample sizes

In RCTs of ultra-orphan drugs, the statistical power may not be sufficient. Although a significance level of 5% and a power of 80–90% are typically used for regular drugs, a reduced power may be needed for ultra-orphan drugs, especially when the number of patients with the target disease is very limited. In some cases, the efficacy and safety of an ultra-orphan drug can be evaluated only with a small sample size that is determined on the basis of the size of the target population and the feasibility of the clinical trial.

#### Post-marketing data collection

To increase the level of evidence, it is useful to collect efficacy and safety data through the pharmacovigilance activities (e.g., post-marketing surveillance) proposed in risk management plans, because clinical trial data have limitations. In addition, clinical trials of ultra-orphan drugs are likely to produce fewer data compared to orphan drugs. Therefore, post-marketing surveillance is more important for ultra-orphan drugs.

There are effective strategies for increasing the level of evidence after marketing: for example, strengthening the collection of spontaneous reports in early post-marketing-phase risk minimization and vigilance, and post-marketing surveys covering all patients. Post-marketing clinical trials can be conducted to assess the recommended doses when dose selection was insufficient in pre-marketing clinical trials, and they can be used to evaluate drug efficacy and safety in patients who do not meet the inclusion criteria used in the pre-marketing clinical trials. No-treatment controls and other controls should be used in post-marketing surveillance for increasing the level of evidence after marketing. It is desirable that external data are available for post-marketing surveillance. The establishment of patient registry systems would be useful not only for developing ultra-orphan drugs but also for increasing the level of evidence of drugs after their marketing has been initiated.

## Discussion

The clinical data packages of the ultra-orphan drugs and the orphan drugs approved in Japan from January 2001 to December 2014 were investigated. We examined the existing recommendations for the efficient assessments of the efficacy and safety of ultra-orphan drugs in clinical trials. The development of ultra-orphan drugs required various arrangements in evidence collection, data source and the clinical trial design. We created “Draft Guidance on the Clinical Development of Ultra-Orphan Drugs” as part of Japan’s Health and Labour Science Research. This draft guidance contains quite concrete content compared to the corresponding guidance issued in the EU and the U.S., and the Japanese draft guidance provides the section on data sources and post-marketing surveillance which the EU guideline and the U.S. guidance do not provide, for the following reasons:.

1. Japanese data alone may be insufficient to explain the efficacy and safety of ultra-orphan drugs. Non-Japanese data and supportive data were used to reinforce them for many ultra-orphan drugs.
2. Post-marketing surveillance is useful to complement a drug’s efficacy and safety data. In fact, post-marketing surveillance of all patients were required in more than 80% of ultra-orphan drugs and post-marketing clinical trials were required in some ultra-orphan drugs.

Regarding clinical trial designs, various arrangements were devised for ultra-orphan drugs. Clinical endpoints were not used as the primary efficacy endpoint of the pivotal trial in almost all ultra-orphan drugs. Single-arm trials were used for efficacy evaluations as pivotal trials, especially for ultra-orphan drugs developed only in Japan. Sample sizes have been determined on the basis of feasibility for some ultra-orphan drugs. Based on these results, we include efficacy endpoint, controls, designs and sample sizes setting in Japanese draft guidance as the contents to be considered in clinical trial designs with a small number of subjects. In contrast, the EU guideline lists specific design of trials, such as response-adaptive methods, sequential designs and n-of-1 trials [11]. Recently, such trial designs for small clinical trials have been investigated [20–24].

N-of-1 trials are considered to be one of the useful trial designs for ultra-orphan drugs with a small number of patients since it directly estimates the curative effect in the individuals and is used for the decision of the appropriate treatment policy. Furthermore, combining the results using techniques from meta-analysis can help estimate the average treatment effect and heterogeneity of treatment effects in the population as well as the individual treatment effects for single patients [20–22, 25–27].

Adaptive deigns and Sequential designs have the advantage to enable to change the design during the trials based on an early results and interim analysis [20–23].

A case in which an adaptive design was used for regulatory submission in Japan is latanoprost/timolol (brand name: Xalacom, indications: glaucoma, hypertonia oculi). For indacaterol (brand name: Onbrez, indication: chronic obstructive pulmonary disease) the dose setting, efficacy and safety were evaluated in a seamless phase II/III study. These drugs are not orphan drugs. The advantages of such an adaptive design are efficient drug development by reducing the development period and the possibility of reducing exposure to a treatment that is not valid or safe for the subjects. However, it is important to understand that a certain number of cases are necessary.

There is little experience with these trial designs in orphan drugs of Japanese clinical trials, but it is thought that these should be positively adopted in future. In the case that these designs are used actually, these will be necessary to prepare clinical trials that can secure high-level evidence using these designs after a good understanding of the characteristics of the disease is obtained and sufficient discussions are held.

On the other hand, there is a type of RCT called a “phase 2.5 trial” as one of the protocol designs of phase II trials in anti-cancer drugs. [28] The purpose of a phase 2.5 trial is a comparison with a control group, but it is not a confirmatory trial. Therefore, surrogate endpoints and significance levels greater than the 5% are allowed. Such approaches could also be used for the development of ultra-orphan drugs and are introduced in Japanese draft guidance.

It is often difficult to conduct clinical trials that aim to confirm the efficacy and safety not only for ultra-orphan drugs but also for regenerative medical products. The reasons are difficulty for securing enough patients and for conducting a comparative study due to the invasiveness of administration. At the present time, four cellular and tissue-based products are approved in Japan, but all of the trials included in these products’ clinical data package as evaluation data were open-label, uncontrolled trials. For the product known as human (autologous) skeletal myoblast-derived cell sheet (brand name: Heart Sheet, indication: serious heart failure), which received conditional and time-limited approval in Japan in 2015, the true endpoint with the seven patients in a Japanese clinical trial were compared with that of 21 patients selected from a database of 112 patients at a specific hospital as external controls. Post-marketing surveillance including no treatment group was also required. Such devised methods could also be used for ultra-orphan drug development and we reflected them in our guidance.

As noted earlier, rare diseases present two basic problems: the limited patient populations hinder the recruitment of sufficient numbers of trial subjects, and the disease pathophysiology is often not elucidated. It is thus difficult to determine the items necessary for diagnosis and drug efficacy evaluations. A natural history study is important to grasp the disease pathophysiology, and it is important to create patient registries for natural history data collection. As described in the FDA’s draft guidance and the EMA guidelines, the expansion of patient registries will be helpful in grasping the numbers of affected patients and designing clinical trials, as well as for use of the registries as historical controls in comparative trials. Following the registered patients can also be done to increase the data of post-marketing surveillance. These are described in “control section” and “post marketing data collection section” of the Japanese draft guidance.

Patient registries are actively implemented in the EU and the U.S., and mainly patients’ organizations are building these registries, such as the Orphanet [29] in the EU. The patient organizations in Japan are not aggressive compared to those in Western countries. Patient registries should be prepared in cooperation with specialized medical institutions, companies, and patient groups. In fact, Japan’s MHLW launched a study group known as “Research for establishing a system for subjecting patient support organizations to voluntary implementation of intractable disease research support” [30] as part of the country’s research on Measures for Intractable Diseases in 2013. A patient registry in Japan for distal myopathy, relapsing polychondritis, Silver-Russell syndrome and Marfan syndrome, i.e., J-RARE.net, has started, and further improvement of patient registries is expected in the future. MHLW and each of Japan’s National Centers are building the clinical development infrastructure that efficient clinical trials can be conducted using patient registration information. Such approaches will contribute to the elucidation of the natural histories of many diseases and the numbers of patients affected, and they can be used to supply historical controls in the development of ultra-orphan drugs.

In addition to the contents described in this draft guidance, if the development of ultra-orphan drugs progresses with the expansion of patient registries, the number of treatment options will increase, and many patients suffering from rare diseases can be helped. In addition, the recognition of rare and very rare diseases will increase and the opportunities for potential patients to be discovered will be strengthened.

## Conclusion

The development of ultra-orphan drugs requires various arrangements in evidence building (RCTs, dose-response trials and extension trials), data source (non-Japanese clinical trial data, multinational trials and supportive data) and the clinical trial design (efficacy endpoints, controls and determination of sample sizes). We created “Draft Guidance on the Clinical Development of Ultra-Orphan Drugs”. The Draft Guidance provided herein will be useful for the future development of ultra-orphan drugs in Japan.

## References

[1] Dolgin E (2010). Big pharma moves from ‘blockbusters’ to ‘niche busters’. Nat Med.

[2] Torres C (2010). Rare opportunities appear on the horizon to treat rare diseases. Nat Med.

[3] FDA (1983). Federal regulations 21CFR part 316.

[4] Regulation (EC) no 141/2000 on orphan medicinal products. 1999. http://eur-lex.europa.eu/LexUriServ/LexUriServ.do?uri=OJ:L:2000:018:0001:0005:en:PDF. Accessed 20 Dec 2016.

[5] Braun MM (2010). Emergence of orphan drug in the United States: a quantitative assessment of the first 25 years. Nat Rev Drug Discov.

[6] Cote T (2010). Orphan products: an emerging trend in drug approvals. Nat Rev Drug Discov.

[7] Melnikova I (2012). Rare diseases and orphan drugs. Nat Rev Drug Discov.

[8] Robert J, Vittorio B (2013). Orphan drugs, orphan disease. The first decade of orphan drug legislation in the EU. Eur Clin Pharmacol.

[9] National Institutes of Biomedical Innovation, Health and Nutrition. Orphan drug, orphan medical device development support program. http://www.nibio.go.jp/part/promote/orphan_support/. Accessed 20 Dec 2016.

[10] Murakami M. Matched analysis on orphan drug designations and approvals: cross regional analysis in the United States, the European Union, and Japan. Drug Discov Today. 2016;21(4):544–9. 10.1016/j.drudis.2016.02.016 26945941

[11] Guideline on Clinical Trials in Small Populations (London, 2006, Doc. Ref. CHMP/EWP/83561/2005). 2005; http://www.ema.europa.eu/docs/en_GB/document_library/Scientific_guideline/2009/09/WC500003615.pdf. Accessed 20 Dec 2016.

[12] FDA. Rare Diseases: Common Issues in Drug Development Guidance for Industry. 2015; http://www.fda.gov/downloads/Drugs/GuidanceComplianceRegulatoryInformation/Guidances/UCM458485.pdf. Accessed 20 Dec 2016.

[13] Regulation (EC) No 847/2000: Laying down the provisions for implementation of the criteria for designation of a medicinal product as an orphan medicinal product and definitions of the concepts ‘similar medicinal product’ and ‘clinical superiority’. 2000; http://ec.europa.eu/health/files/eudralex/vol-1/reg_2000_847/reg_2000_847_en.pdf. Accessed 20 Dec 2016.

[14] Recommendation (EU) 2009/C 151/02: On an action in the field of rare diseases. 2009; http://eur-lex.europa.eu/LexUriServ/LexUriServ.do?uri=OJ:C:2009:151:0007:0010:EN:PDF. Accessed 20 Dec 2016.

[15] Ministry of Health, Labour and Welfare, Japan. Overview of the orphan drug/medical device designation system. 1993; (Published in Japanese). http://www.mhlw.go.jp/general/seido/iyaku/kisyo/.

[16] Health Science Council: Disease control part meeting incurable disease control committee. Measures to counter intractable diseases (recommendation). 2013; (Published in Japanese) http://www.mhlw.go.jp/stf/shingi/2r9852000002udfj-att/2r9852000002udh0.pdf.

[17] Patients Association for Distal Myopathies (PADM). (Published in Japanese). http://npopadm.com. Accessed 20 Dec 2016.

[18] Kenketsu Venilon-I, Review report. 2010; (Published in Japanese). http://www.pmda.go.jp/drugs/2010/P201000017/20001100_22100AMX01040_A100_1.pdf.

[19] In their notification entitled “Points to Be Considered by the Review Staff Involved in the Evaluation Process of New Drug”. 2008; (Published in Japanese). https://www.pmda.go.jp/files/000157674.pdf. Accessed 20 Dec 2016.

[20] Gupta S (2011). A framework for applying unfamiliar trial designs in studies of rare diseases. J Clin Epidemiol.

[21] Cornu C (2013). Experimental designs for small randomised clinical trials: an algorithm for choice. Orphanet J Rare Dis.

[22] Nony P (2014). A methodological framework for drug development in rare diseases. Orphanet J Rare Dis.

[23] Chow SC (2008). Adaptive design methods in clinical trials – a review. Orphanet J Rare Dis.

[24] Lilford RJ (1995). Clinical trials and rare diseases: a way out of a conundrum. BMJ.

[25] Schork NJ (2015). Personalized medicine: time for one-person trials. Nature.

[26] Agency for Healthcare Research and Quality (2015). Design and implementation of N-of-1 trials: a user’s guide.

[27] Duan N (2013). Single-patient (n-of-1) trials: a pragmatic clinical decision methodology for patient-centered comparative effectiveness research. J Clin Epidemiol.

[28] Rubinstein L, Crowley J, Ivy P (2009). Randomized phase II designs. Clin Cancer Res.

[29] Orphanet. http://www.orpha.net/consor/cgi-bin/index.php. Accessed 22 Dec 2016.

[30] Health Labour Sciences Research Grant, Incurable disease conquest study project, “Research for establishing a system for subjecting patient support organizations to voluntary implementation of intractable disease research support”, Research report. 2012–2013; (Published in Japanese). http://www.nanbyo.jp/kenkyu/hokoku/H25siryo/24-25sogohokoku.pdf. Accessed 22 Dec 2016.

